# Who Decides? Balancing Autonomy and Safety in Irish Psychiatry under the Mental Health Bill 2024

**DOI:** 10.1192/j.eurpsy.2026.10756

**Published:** 2026-07-31

**Authors:** S. Albarzani, A. K. Rios-Landeo, I. I. Abdelaziz, M. Dhuhaibawi, O. Akpubi

**Affiliations:** Psychiatry, University Hospital of Waterford, Waterford, Ireland

## Abstract

**Introduction:**

The Mental Health Bill 2024 introduces a landmark change in Irish mental health law, making advance directives legally binding except through High Court intervention.^1^ While enhancing patient autonomy, the Bill raises important ethical and clinical challenges, particularly in balancing individual choice with psychiatrists’ duty to safeguard wellbeing.^1,2^

**Objectives:**

To explore psychiatrists’ perspectives on familiarity with the Bill, overall attitudes, anticipated impact on clinical practice, workload implications, and support for its implementation.

**Methods:**

We conducted a cross-sectional observational survey of psychiatrists across multiple regions in Ireland. Participation was anonymous and voluntary, with implied informed consent. No identifiable data were collected, ensuring strict confidentiality and adherence to ethical standards. Structured multiple-choice questions were used to capture a broad range of professional perspectives.

**Results:**

Thirty clinicians completed the survey. Most were psychiatrists (56.7%), with 30.0% at Senior House Officer/Intern level and 13.3% at Registrar/Specialist Registrar level. Experience ranged from <3 years (23.3%) to >15 years (23.3%), with 26.7% each having 3–7 or 8–15 years.

Familiarity with the Bill was moderate: 33.3% were very familiar, 53.3% somewhat familiar, and 13.3% not familiar (Figure 1). Overall views were negative in 50.0%, neutral in 43.3%, and positive in 6.7% (Figure 2).

Regarding clinical impact, 66.7% felt the Bill would not improve practice, 20.0% expected no change, and 13.3% anticipated improvement (Figure 3). On patient rights, 46.7% felt protections were partial, 33.3% inadequate, and 20.0% adequate. For staff workload, 80.0% reported no benefit, 16.7% some benefit, and 3.3% clear benefit.

Support for implementation in its current form was limited: 46.7% opposed, 46.7% favoured partial adoption with amendments, and 6.7% supported full adoption.

**Image 1:**
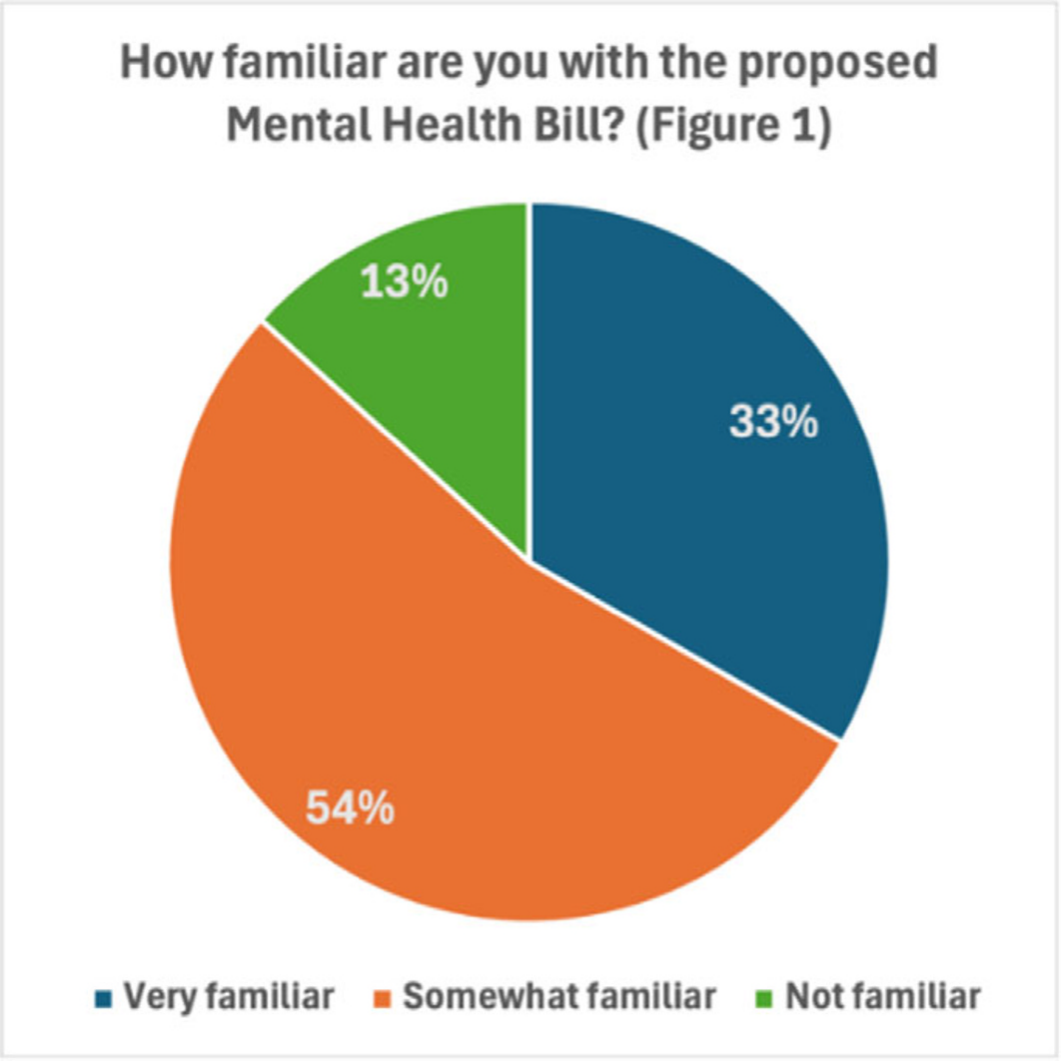

**Image 2:**
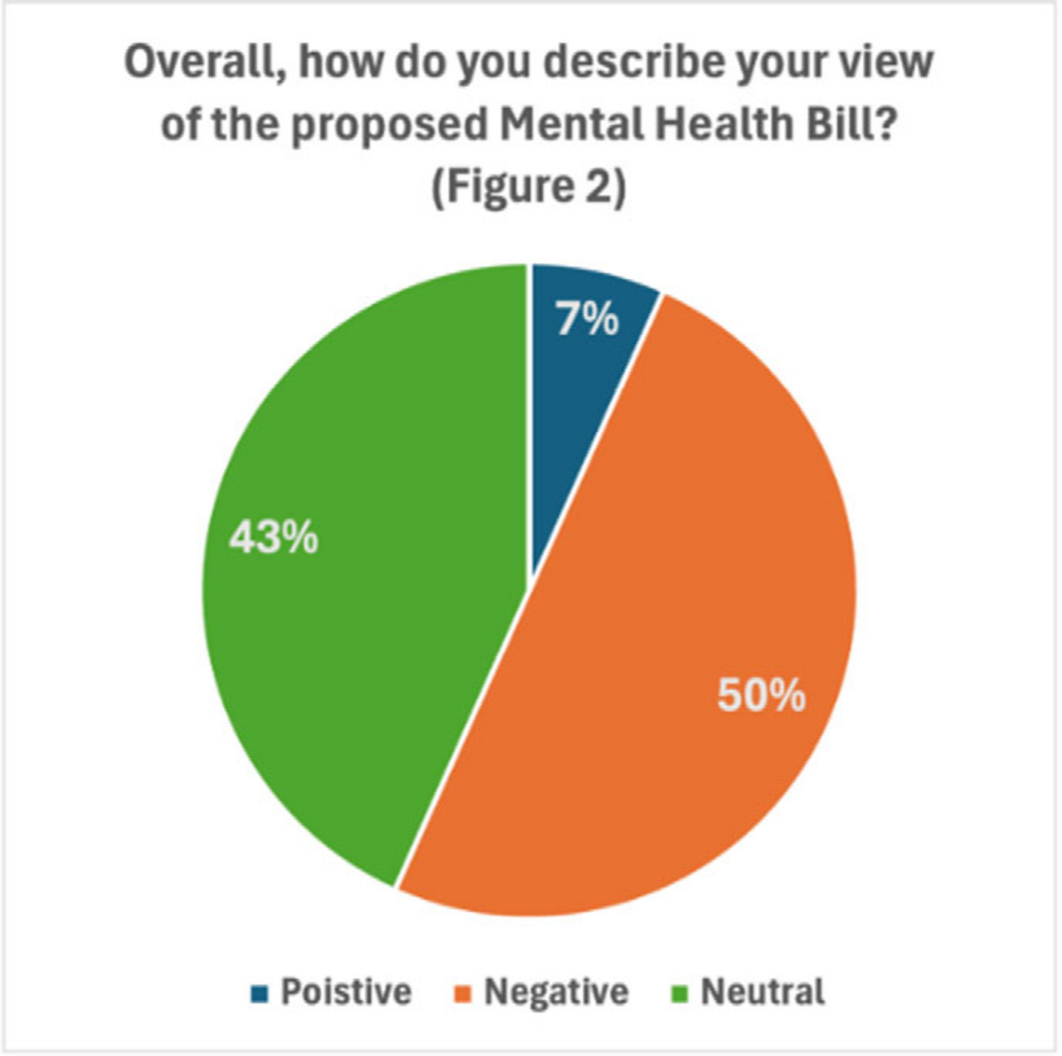

**Image 3:**
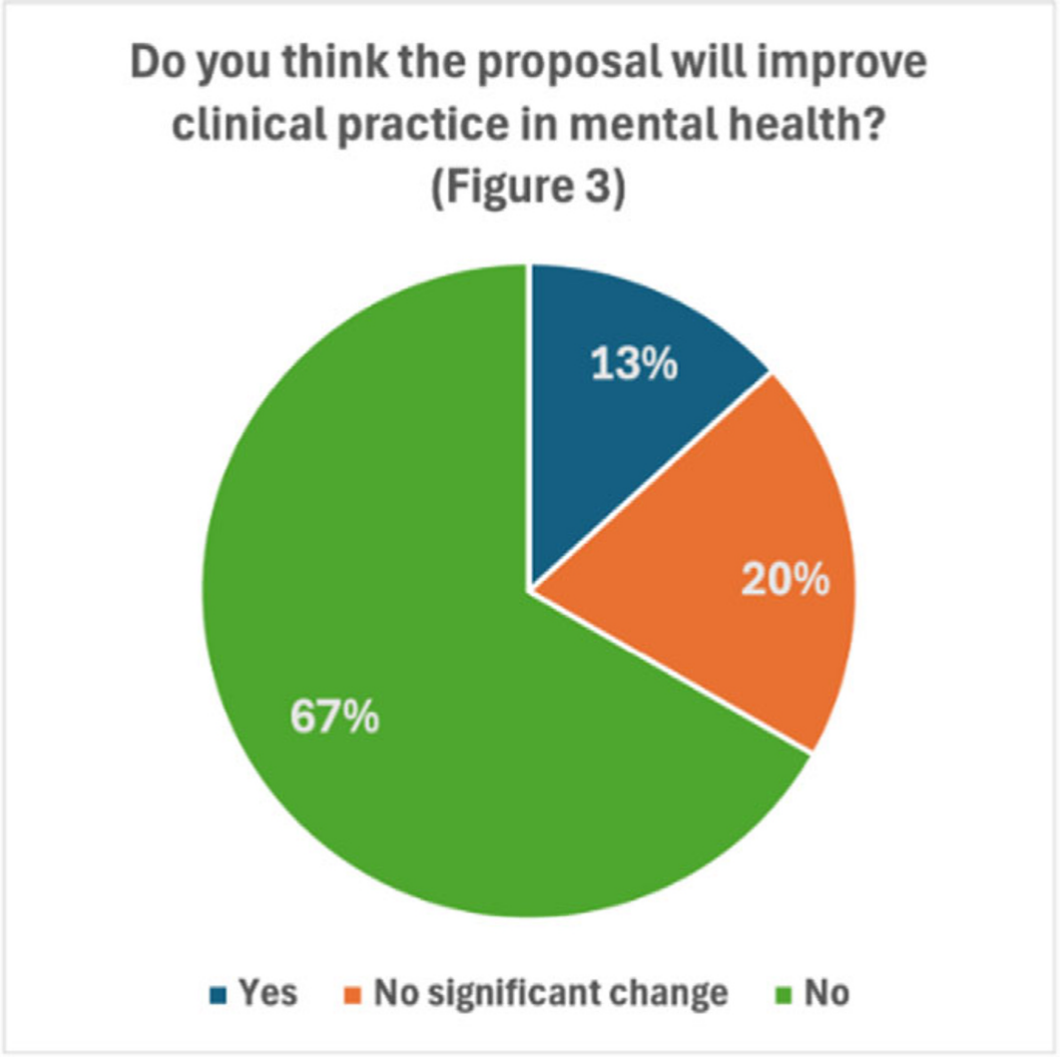

**Conclusions:**

The findings highlight professional caution and ethical tension between autonomy and safety. While the Bill strengthens patient rights, psychiatrists foresee practical and operational challenges, limited clinical benefit, and conditional support for implementation. These results emphasize the need for structured guidance, robust ethical frameworks, and clinician engagement to navigate complex decision-making, reconcile patient-centred care with operational realities, and ensure reforms are implemented responsibly. The Bill presents an opportunity to advance Irish psychiatric practice, but success will require careful, ethically informed, and collaborative implementation.

**References:**

1. Kelly BD. *Ir J Med Sci.* 2020;189:1127-34.
2. Kelly BD. *Ir J Med Sci.* 2024;193:2897-2914.

**Disclosure of Interest:**

None Declared

